# Disparities in Medical Debt Among U.S. Adults with Serious Psychological Distress

**DOI:** 10.1089/heq.2020.0090

**Published:** 2020-12-30

**Authors:** Priscilla J. Novak, Mir M. Ali, Maria X. Sanmartin

**Affiliations:** ^1^ Department of Health Policy and Management, School of Public Health, University of Maryland at College Park, College Park, Maryland, USA.; ^2^ Department of Health Professions, Hofstra University, Hempstead, New York, USA.

**Keywords:** medical debt, disparities, insurance

## Abstract

**Purpose:** To examine indebtedness for medical care among racial and ethnic minorities and people with serious psychological distress (SPD) using a nationally representative sample in the United States.

**Methods:** Using the 2014–2017 Medical Expenditure Panel Survey, we examine medical debt among individuals with SPD. We develop a logistic regression model to estimate the odds of medical debt by SPD status. We stratify the odds of medical debt for those with SPD by insurance type.

**Results:** The results indicate that after controlling for predisposing, enabling, and physical needs factors, those experiencing SPD have double the odds of having medical debt compared with those without SPD. Non-Hispanic blacks had higher odds of medical debt compared with non-Hispanic whites. We find that individuals with SPD covered under private health insurance have double the odds of having medical debts; and those who are uninsured have triple the odds of having medical debt compared with their counterparts without SPD.

**Conclusion:** The findings suggest that odds of medical debt are higher among people with SPD, even when insured. Additional health policy initiatives to address medical debt among those with SPD may be warranted.

## Introduction

Before the passage of the Affordable Care Act (ACA) in 2010, one of the benefits suggested by advocates of health insurance expansion was a hypothesized increase in access to care and reduction in medical debt if more Americans had health insurance.^1,2^ Findings post-ACA indicate that health insurance coverage was expanded^3–5^; however, high out-of-pocket costs continue to create barriers to accessing needed health care.^6–10^ Furthermore, the literature on personal bankruptcy filings suggests that medical conditions continue to be a driver of personal bankruptcy filings.^11–13^ Although the relationship between medical bills and bankruptcy has been studied, fewer studies have been conducted on medical debt. Medical debt may constitute a barrier to appropriate care seeking. However, cost- and debt-related barriers to accessing health care are not evenly distributed and several studies suggest that people with serious psychological distress (SPD) have greater difficulty accessing needed health care.^14,15^

Many of the available studies on medical debts assess care seeking and persistent debts among people who have survived cancer, with the preponderance of evidence suggesting that cancer survivorship is associated with persistent financial hardship and medical debts.^16–18^ Additional studies have examined the odds of filing for bankruptcy after debilitating spinal cord injuries,^19^ the relationship between personal debt and suicidal ideation,^20^ and the relationship between medical debt and use of payday loans.^21^ These studies suggest that there are deleterious influences of having medical debts, including postponing needed care because of a desire to not have any more bills that cannot be paid.

Annually, between 3.5% and 5% of the U.S. adult population experiences SPD.^22^ Research on services utilization among U.S. adults with SPD suggests that the ACA increased needed mental health service utilization.^23,24^ Furthermore, having health insurance may decrease anxiety among low-income urban women.^25^ The literature suggests that individuals with SPD have higher utilization of physical health care services than those without SPD,^14,15,26^ and this includes higher numbers of emergency department visits^27^ and poorer health outcomes after surgery.^28^ Although health services utilization are necessary for individuals with SPD, it may be that their high levels of utilization and poorer outcomes after interventions drive high spending and medical debts.

SPD has been reported to increase barriers to receiving adequate health care by increasing care co-ordination challenges.^29–33^ Given the siloed nature of mental and physical health care delivery in the United States, prior studies indicate that people with SPD have to navigate and pay for care across physical and mental health care providers.^34,35^ Although higher expenditures have been documented for people with SPD,^36,37^ little is known about medical debt among this population. The objective of this study is to examine the impact of SPD on medical debt in a nationally representative sample of U.S. adults.^6,38^ We hypothesize that individuals with SPD will be disproportionately represented in the group with medical debt; and that people with SPD would have higher odds of reporting medical debt compared with people without SPD. This topic is of policy significance as one of the main aims of expanding health insurance coverage was to reduce financial hardship associated with utilization of needed health services.

## Methods

### Participants and procedures

We use data from the 2014–2017 Medical Expenditure Panel Survey (MEPS), public use household file. The MEPS is collected and produced by the Agency for Healthcare Research and Quality and contains information on demographics, health insurance coverage, health care use, and health care expenditures among the U.S. civilian noninstitutionalized population. The Agency for Healthcare Research and Quality uses the Chesapeake Institutional Review Board for review of its human subjects protections. All MEPS respondents provide informed consent and are free to refuse to participate in the survey without fear of retribution. Completion of the survey ranges between 70% and 80%.^39^ The MEPS uses a complex sampling design and develops survey weights to make the sample representative of the national population. Survey weights were used in all analysis. This research was deemed exempt by the IRBs of our institutions.

### Conceptual model and control variables

The U.S. civilian noninstitutionalized adults aged 18–64 years are the subjects of this study. We combine 2014–2017 data using the pooling procedure provided by the MEPS. We use the Kessler-6 score in the MEPS to construct a binary indicator for SPD. The Kessler-6 screener contains six questions such as, “During the past 30 days, about how often did you feel depressed?”^40^ and, “During the past 30 days, about how often did you feel hopeless?.” The answers use the categories “all of the time,” “most of the time,” “some of the time,” “a little of the time,” or “never.”^41^ The survey then asks, “Taking them altogether, did these feelings occur “More often in the past 30 days than is usual for you,” “about the same as usual,” or “less often than usual?” Scores can range between 0 and 24. People scoring 13 or more are classified as having SPD and those scoring 12 or less are classified as not having SPD.^26^ SPD is not itself a diagnosis, but rather serves as an indicator of distress that is serious enough to warrant additional evaluation by a health care professional.^42,43^

### Measures and outcomes

The binary variable medical debt is based on respondents' answers to the question on “having any medical bills that you are unable to pay at all?” People who indicated that there are bills that they are unable to pay are categorized as having medical debt, and those who responded that this was not the case are categorized as not having medical debt.

We use the Andersen model of health services utilization to select covariates in our model.^44^ The selected covariates include the predisposing characteristics of age, gender (male or female), marital status (currently married or not), and race/ethnicity (non-Hispanic white, non-Hispanic black/African American, Hispanic, Asian American, and other races). The enabling characteristics include educational attainment (less than high school, high school, some college, college, or advanced degree) income level (poor, near poor, low income, middle income, or high income), region of residence (northeast, south, midwest, or west), insurance type (private insurance, Medicaid, or uninsured). Finally, for the needs characteristics, we use self-reported health status (excellent, very good, good, fair, and poor).

### Statistical analysis

We first develop descriptive statistics and examine the population characteristics of our sample by stratifying by medical debt status. Among those with SPD, we examine the proportions with medical debt over the 4-year period that this study covers. Then, we estimate a logistic regression model to estimate the odds of medical debt when an individual has SPD. Finally, we stratify the models by health insurance type (private insurance, Medicaid, or uninsured) to examine how the impact of SPD on the odds of having medical debt varies by individual's health insurance coverage status. We used Stata 14 to conduct all analysis.

## Results

Table 1 presents demographic characteristics of the respondents in our analysis by their medical debt status. Our analytic sample consists of 78,918 (weighted *N*=193 million) adults, out of whom 5531 (weighted *N*=11,363,469) reported having medical debt. There was a higher proportion of respondents with SPD among those with medical debt (0.20 vs. 0.05; *p*<0.000). By race and ethnicity, non-Hispanic white and Asian American adults represented a lower proportion of those with medical debts, whereas non-Hispanic blacks, Hispanics, and multiracial individuals had higher proportions of medical debt (*p*<0.000 for all categories). The proportions of medical debt by education status revealed that those with high school education or less had the highest proportion of medical debt and those with college degree (0.09) and advanced degrees (0.02) had the lowest proportion (*p*<0.000 for all categories). Poor (0.22), low-income (0.22), and middle-income (0.34) groups had higher proportion of medical debt compared with the high-income group (0.14). Those who were not married had a higher proportion of medical debt (58% vs. 42%; *p*<0.000).

**Table 1.** Demographic Characteristics of U.S. Adults by Medical Debt Status, Combined Medical Expenditure Panel Survey Data, 2014–2017

	Medical debt	No medical debt
	Weighted proportions (std. error)	Weighted proportions (std. error)
SPD		
Yes	0.20 (<0.01)^***^	0.05 (<0.01)
No	0.80 (<0.01)^***^	0.95 (<0.01)
Self-rated physical health		
Excellent	0.14 (0.01)^***^	0.28 (0.01)
Very good	0.23 (0.01)^***^	0.36 (0.01)
Good	0.33 (0.02)^***^	0.27 (0.01)
Fair	0.20 (0.01)^***^	0.08 (0.01)
Poor	0.10 (0.03)^***^	0.02 (0.01)
Age		
18–25 years	0.15 (0.01)	0.15 (<0.01)
26–35 years	0.21 (0.01)	0.22 (<0.01)
36–45 years	0.19 (0.01)	0.20 (<0.01)
46–55 years	0.23 (0.01)^*^	0.22 (<0.01)
56–64 years	0.21 (0.01)	0.20 (<0.01)
Race/ethnicity		
Non-Hispanic white	0.54 (0.02)^***^	0.61 (0.01)
Non-Hispanic black	0.20 (0.01)^***^	0.12 (0.01)
Hispanic	0.20 (0.01)^**^	0.17 (0.01)
Asian	0.02 (<0.01)^***^	0.06 (<0.01)
Non-Hispanic Other	0.05 (0.01)^***^	0.03 (0.01)
Marital status		
Married	0.42 (0.01)^***^	0.53(0.04)
Not currently married	0.58 (0.03)^***^	0.47 (0.01)
Education		
<High school	0.20 (0.01)^***^	0.12 (0.01)
High school	0.37 (0.01)^***^	0.28 (0.01)
Some college	0.32 (0.01)^***^	0.28 (0.01)
College	0.09 (0.01)^***^	0.20 (0.01)
>4 Years of college	0.02 (0.01)^***^	0.12 (0.01)
Health insurance		
Private insurance	0.51 (0.03)^***^	0.75 (0.01)
Public insurance	0.28 (0.01)^***^	0.14 (0.01)
Uninsured	0.21 (0.02)^***^	0.11 (0.01)
Income		
Poor	0.22 (0.01)^***^	0.11 (0.01)
Near poor	0.08 (0.01)^***^	0.03 (0.01)
Low income	0.22 (0.01)^***^	0.11 (0.01)
Middle income	0.34 (0.01)^***^	0.29 (0.01)
High income	0.14 (0.01)^***^	0.46 (0.01)
*N*	5531	73,387
Weighted *N*	13.4 million	193 million

Source: Authors' analysis of data for 2014–2017 from the Medical Expenditure Panel Survey.

*N*=78,918 MEPS respondents, of whom 5531 stated that they had medical debts that they could not pay at all. The proportions are weighted using survey weights to make the results nationally representative.

^*^ Significant at *p*<0.05 confidence level.

^**^ Significant at *p*<0.005 confidence level.

^***^ Significant at *p*<0.001 confidence level.

MEPS, Medical Expenditure Panel Survey; SPD, serious psychological distress.

Among individuals with medical debt, 51% had private insurance (vs. 75% among those without medical debt), 28% had public insurance (vs. 14% among those without medical debt), and 21% were uninsured (vs. 11% among those without medical debt). Finally, among those with medical debt 14% reported to be in excellent health and 23% reported to be in very good health.

**FIG. 1.**
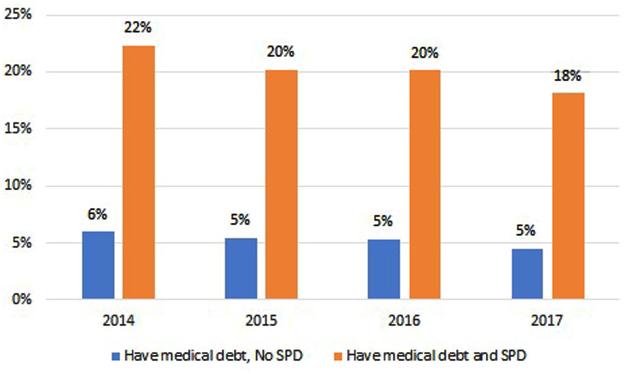
Medical debt by SPD status among adults aged 18–64 years, 2014–2017. Source: Authors' analysis of data for 2014–2017 from the Medical Expenditure Panel Survey. *N*=2908 people with SPD. The proportions are weighted using survey weights to make the results nationally representative. “Medical debt” refers to respondents who reported that they had bills that they could not pay at all. Differences between those with and without SPD are significant at the *p*<0.001 confidence level across all 4 years. SPD, serious psychological distress.

From Figure 1, we see that proportion of individuals with SPD had a consistent and higher prevalence of medical debt compared with their counterparts without SPD. Specifically, in 2014, 22% of those with SPD had medical debt compared with 6% of individuals without SPD. The proportions were constant over the years with 20% versus 5% in 2015, 20% versus 5% in 2016, and 18% versus 5% in 2017.

Table 2 reports estimates from logistic regression model examining the correlation between SPD and the odds of having medical debt. The results indicate that compared with individuals without SPD, individuals with SPD had higher odds (odds ratio [OR] 1.99; *p*<0.000) of medical debt. Poor self-reported physical health was a strong predictor of medical debt (OR 5.44; *p*<0.000). No significant correlation was observed across the age categories. Females had slightly higher odds of having medical debt (OR 1.07; *p*<0.05). Non-Hispanic blacks (OR 1.24; *p*<0.000) and other race/ethnicity (OR 1.56; *p*<0.000) had higher odds of medical debt, than the non-Hispanic whites. Asian Americans (OR 0.45; *p*<0.000) had lower odds of medical debt compared with non-Hispanic whites.

**Table 2.** Odds of Having Medical Debt for Working Aged U.S. Adults, 2014–2017

	Odds ratio	p > t	95% Confidence interval	
Needs factors				
SPD	1.99	**0.00**	1.71	2.32
Self-report of health (excellent is the comparator)				
Very good	1.25	**0.00**	1.08	1.45
Good	1.99	**0.00**	1.74	2.28
Fair	3.19	**0.00**	2.76	3.68
Poor	5.44	**0.00**	4.56	6.48
Predisposing factors				
Age (18–29 years is the comparator)				
30–44 years	1.00	0.96	0.88	1.14
45–54 years	1.07	0.26	0.95	1.21
54–65 years	0.94	0.37	0.82	1.08
Female	1.07	**0.04**	1.00	1.14
Not married	1.05	0.40	0.94	1.17
Race/ethnicity (non-Hispanic white is the comparator)				
Non-Hispanic black	1.24	**0.00**	1.08	1.41
Hispanic	0.90	0.24	0.76	1.07
Asian American	0.45	**0.00**	0.31	0.63
Other race/ethnicity	1.56	**0.00**	1.21	2.01
Enabling factors				
Education (less than high school is the comparator)				
High school	1.00	0.97	0.89	1.13
Some college	0.99	0.92	0.86	1.15
College	0.60	**0.00**	0.48	0.74
Advanced degree	0.36	**0.00**	0.26	0.49
Insurance (private insurance is the comparator)				
Public insurance	1.05	0.48	0.92	1.21
Uninsured	1.56	**0.00**	1.35	1.82
Income category (poor is the comparator)				
Near poor	1.24	**0.03**	1.02	1.52
Low income	1.28	**0.00**	1.12	1.46
Middle income	0.95	0.49	0.82	1.10
High income	0.37	**0.00**	0.30	0.46
Region (northeast is the comparator)				
Midwest	1.16	0.27	0.89	1.51
South	1.38	**0.00**	1.11	1.70
West	0.74	**0.01**	0.59	0.94
Year (2014 is the comparator)				
2015	0.93	0.33	0.81	1.08
2016	0.94	0.42	0.81	1.09
2017	0.80	**0.01**	0.68	0.95

Bold text indicates statistically significant results.

Source: Authors’ analysis of data for 2014–2017 from the Medical Expenditure Panel Survey.

*N*=78,297. Survey weights were used to make the results nationally representative.

Across the enabling characteristics, those who had completed college (OR 0.60; *p*<0.000) or with more than a 4-year college degree (OR 0.36; *p*<0.000) had lower odds of medical debt. Compared with individuals <100% of the Federal Poverty Level (FPL), those who were near poor (i.e., 101–138% of the FPL; OR 1.24; *p*<0.05) and low income (i.e., 139–200% of the FPL; OR 1.28; *p*<0.000) had higher odds of medical debt. Those with high incomes (i.e., >400% of the FPL; OR 0.37; *p*<0.000) had lower odds of medical debt. Respondents living in the south (OR 1.38; *p*<0.000) had higher odds of medical debt, whereas those residing in the west (OR 0.75; *p*<0.001) had lower odds of medical debt, compared with residents of the northeast.

In Table 3, we stratify our logistic regression by health insurance type status. Among those who were uninsured, having SPD was associated with approximately three times the odds of having a medical debt (OR 2.96; *p*<0.000). Among those with private insurance, the odds ratio for medical debt among those with SPD was 2.23 (*p*<0.00). Finally, for those with Medicaid, the odds ratio for medical debt among those with SPD was 1.56 (*p*<0.00).

**Table 3.** Odds Ratio of Medical Debt by Health Insurance Status

	Uninsured (CI)	Private insurance (CI)	Medicaid (CI)
SPD	2.96^***^ (2.11–4.15)	—	—
	—	2.23^***^ (1.72–2.88)	—
	—	—	1.56^***^ (1.28–1.91)

Source: Authors' analysis of data for 2014–2017 from the Medical Expenditure Panel Survey.

*N*=78,297. Survey weights were used to make the results nationally representative. Control variables include age, gender, marital status, education, income, insurance coverage, self-reported health status, and survey year.

^***^ Significant at *p*<0.001 confidence level.

CI, confidence interval.

## Discussion

This study provides preliminary evidence on medical debt among U.S. adults aged 18–64 years, finding that individuals with SPD have double the odds of medical debt, with the strongest relationship among those who are uninsured, followed by private insurance, and then Medicaid. The finding that SPD was associated with increased odds of medical debt among those who were uninsured, as well as among those with private health insurance coverage signifies the risk that SPD possess in terms of medical debt and financial instability.

Our stratified model showed that the odds of having medical debt for those with SPD but covered under Medicaid and private insurance is statistically significant, suggesting that there might be a potential problem with benefit design for both Medicaid and private insurance since it is unable to protect individuals with SPD from incurring medical debt. This finding adds to the literature on barriers to health services utilization among individuals with SPD by providing evidence that they not only have difficulty in accessing care, but also are more likely to incur medical debt.^45^ People with SPD have a high level of need for health care services. Plan design may benefit from additional consideration of what services beneficiaries with SPD utilize that result in them having medical debt. Some health systems have begun offering housing,^46^ employment,^47^ and financial literacy^48^ services in an effort to address the social determinants of health among those with SPD, and additional research on how these programs function may inform best practices in population health management.

### Health equity implications

Race and ethnicity had an important relationship with medical debt, with higher odds of medical debt among non-Hispanic blacks and the multiracial ethnic group and lower odds of medical debt among Asians compared with their non-Hispanic white counterparts. In our model, there was a positive correlation between medical debt and being in a low-income group. We hypothesize that this may be because although Medicaid expansion covered low-income individuals, it was not available to those who were near poor but who marginally exceeded the ceiling for Medicaid coverage. We observed that college completion and advanced degree holders had lower odds of medical debt, signifying the potential mitigating impact of education on medical debt. We also observed geographic differences. Those residing in the south had higher odds of medical debt, whereas those living in the west had lower odds of medical debt. This finding is consistent with the fact that many of the states that chose not to expand Medicaid eligibility were located in the south.

We borrow from the stress accumulation model of disease to hypothesize that people with medical debts may experience heightened anxiety, which could increase health inequity. Assuming that the SPD was present first and that the need for health services arose second, one might rightly propose additional measures to promote population mental health. However, our study does not capture the direction of the relationship between SPD and medical debt. If medical debt heightens anxiety, feelings of hopelessness, and feeling “down,” one might correctly propose to target medical debt as the causal factor to be addressed through health policy and programs. We observed little change in the rates of medical debt across 2014–2017. Given that the full implementation of the ACA began in 2014, the MEPS only began collecting data on medical debt in 2014. Additional MEPS data years might provide greater insight into temporal trends in medical debt.

There are some limitations that are worth noting. First, our study was observational and the interpretation of a causal relationship between SPD and medical debt must be undertaken with caution. Second, as in all survey-based research, there is the possibility of recall bias or social desirability influencing answers. Finally, we are unable to control for the amount of the medical debt that people have. The Federal Reserve Board has published data briefs indicating that >30% of American households have <$400 in emergency cash reserves to pay for unexpected expenses.^49^ Thus, we are able to measure the impact of SPD on only the likelihood of incurring a medical debt but not on the amount of medical debt, which might be higher among individuals with SPD compared with their counterparts without SPD.

## Conclusion

This initial study provides preliminary evidence on higher odds of medical debt among U.S. adults with SPD. SPD was associated with statistically significant higher odds of medical debt regardless of the individuals' health insurance status. Policy initiatives to address health insurance coverage and health services utilization might benefit from being cognizant of the financial hardship that SPD imparts on individuals even when they have health insurance. More research is needed to determine effective solutions to enable Americans with SPD to avoid medical debt.

## Abbreviations Used

ACA: Affordable Care Act
CI: confidence interval
MEPS: Medical Expenditure Panel Survey
OR: odds ratio
SPD: serious psychological distress
FPL: Federal Poverty Level
